# Supplementary material for: Leucocyte Telomere Length and Lung Cancer Risk: A Systematic Review and Meta-Analysis of Prospective Studies
Source: Cancers (Basel). 2024 Sep 21;16(18):3218. doi: 10.3390/cancers16183218 (PMC11430440; doi:10.3390/cancers16183218)
Supplement: Supplementary file 1 [file cancers-16-03218-s001.zip › cancers-3155566-supplementary.pdf]

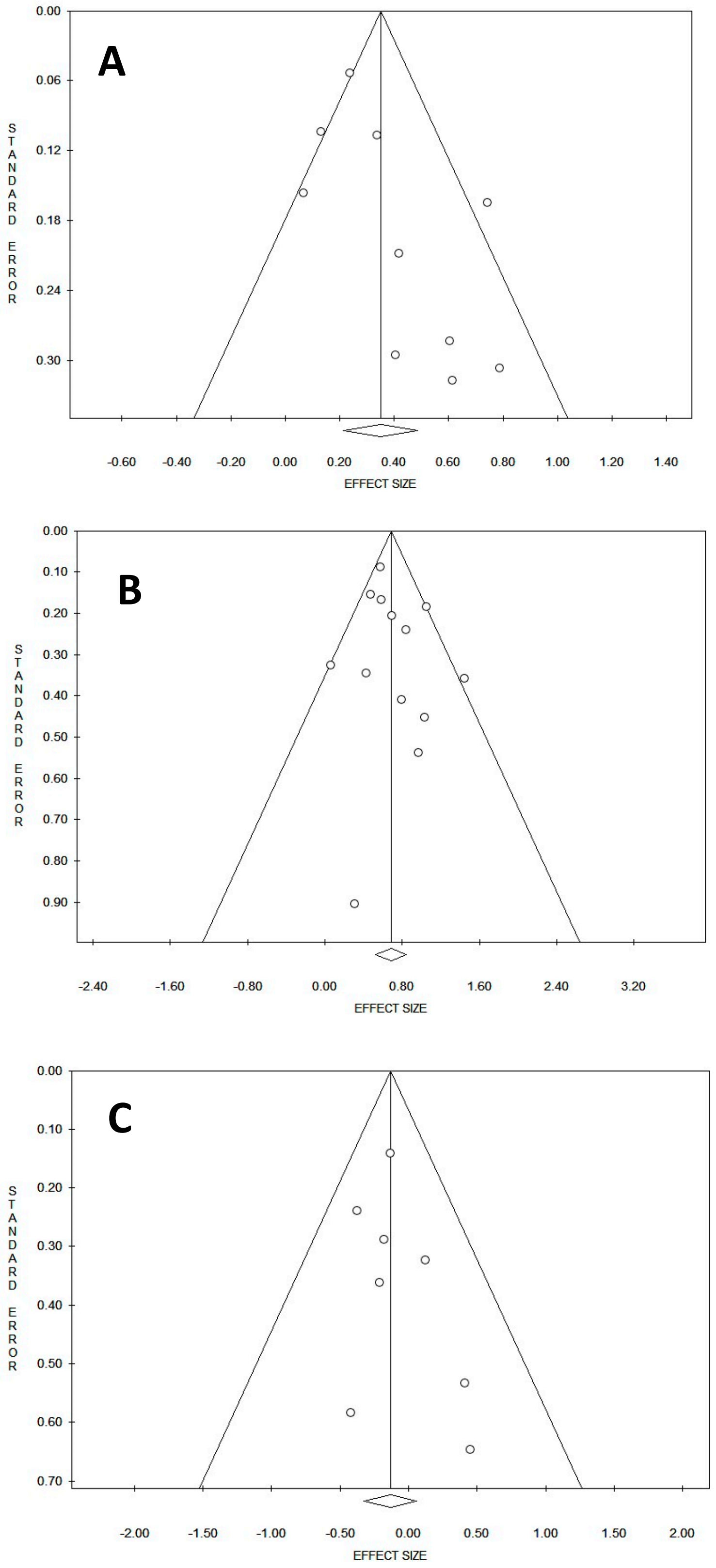

Supplementary Figure S1. Funnel plots of the meta-analysis on all type of lung cancer (**A**), adenocarcinoma (**B**) and squamous cell carcinoma (**C**).

Supplementary Table S1. list of articles excluded from the meta-analysis and reasons for exclusion.

List of articles excluded	Reasons for exclusion (numbers)
Teng et al., 2023 [3]	Genetically predicted LTL by Mendelian randomization (7)
Cortez Cardoso Penha et al., 2023 [6]	
Gao et al., 2020 [9]	
Cao et al., 2019 [10]	
Kachuri et al., 2019 [11]	
Haycock et al., 2017 [14]	
Zhang et al., 2015 [16]	
Shiraishi et al., 2024 [1]	Genetically predicted LTL by SNPs variants (5)
Bhat et al., 2023 [4]	
Chang et al., 2021 [7]	
Rode et al., 2016 [15]	
Machiela et al., 2015 [18]	
Xue et al., 2020 [8]	Retrospective case-control (6)
Sun et al., 2015 [19]	
Sanchez-Espiridion et al., 2014 [20]	
Jang et al., 2008 [24]	
Wu et al., 2003 [25]	
Hosgood et al., 2009 [23]	
Belić et al., 2023 [5]	Not reporting the risk of lung cancer (outcome of interest) (4)
Autsavapromporn et al., 2018 [12]	
Willeit et al., 2010 [22]	
Weischer et al., 2013 [25]	
Doherty et al., 2018 [13]	Lung cancer mortality (1)
Fernández-Marcelo et al., 2015 [17]	DNA not extracted form blood (1)
Córdoba-Lanús et al., 2024 [2]	Considering subjects with COPD (1)

1. Shiraishi, K.; Takahashi, A.; Momozawa, Y.; Daigo, Y.; Kaneko, S.; Kawaguchi, T.; Kunitoh, H.; Matsumoto, S.; Horinouchi, H.; Goto, A.; et al. Identification of telomere maintenance gene variations related to lung adenocarcinoma risk by genome-wide association and whole genome sequencing analyses. *Cancer Commun (Lond)* **2024**, *44*, 287-293.
2. Córdoba-Lanús, E.; Montuenga, L.M.; Domínguez-de-Barros, A.; Oliva, A.; Mayato, D.; Remírez-Sanz, A.; Gonzalvo, F.; Celli, B.; Zulueta, J.J.; Casanova, C. Oxidative damage and telomere length as markers of lung cancer development among chronic obstructive pulmonary disease (COPD) smokers. *Antioxidants (Basel)* **2024**, *13*, 156.
3. Teng, Y.; Huang, D.Q.; Li, R.X.; Yi, C.; Zhan, Y.Q. Association between telomere length and risk of lung cancer in an asian population: a Mendelian randomization study. *World J. Oncol.* **2023**, *14*, 277-284.

4. Bhat, G.R.; Jamwal, R.S.; Sethi, I.; Bhat, A.; Shah, R.; Verma, S.; Sharma, M.; Sadida, H.Q.; Al-Marzooqi, S.K.; Masoodi T.; et al. Associations between telomere attrition, genetic variants in telomere maintenance genes, and non-small cell lung cancer risk in the Jammu and Kashmir population of North India. *BMC Cancer* **2023**, *23*, 874.
5. Belić, M.; Sopić, M.; Roksandić-Milenković, M.; Ćeriman, V.; Guzonijić, A.; Vukašinović, A.; Ostanek, B.; Dimić, N.; Jovanović, D.; Kotur-Stevuljević, J. Correlation of short leukocyte telomeres and oxidative stress with the presence and severity of lung cancer explored by principal component analysis. *Folia Biol. (Praha)* **2023**, *69*, 59-68.
6. Cortez, C.P.R.; Smith-Byrne, K.; Atkins, J.R.; Haycock, P.C.; Kar, S.; Codd, V.; Samani, N.J.; Nelson, C.; Milojevic, M.; Gabriel, A.A.G.; et al. Common genetic variations in telomere length genes and lung cancer: a Mendelian randomisation study and its novel application in lung tumour transcriptome. *Elife* **2023**, *12*, e83118.
7. Chang, X.; Gurung, R.L.; Wang, L.; Jin, A.; Li, Z.; Wang, R.; Beckman, K.B.; Adams-Haduch, J.; Meah, W.Y.; Sim, K.S.; et al. Low frequency variants associated with leukocyte telomere length in the Singapore Chinese population. *Commun. Biol.* **2021**, *4*, 519.
8. Xue, Y.; Guo, X.; Huang, X.; Zhu, Z.; Chen, M.; Chu, J.; Yang, G.; Wang, Q.; Kong, X. Shortened telomere length in peripheral blood leukocytes of patients with lung cancer, chronic obstructive pulmonary disease in a high indoor air pollution region in China. *Mutat. Res. Genet. Toxicol. Environ. Mutagen.* **2020**, *858-860*, 503250.
9. Gao, Y.; Wei, Y.; Zhou, X.; Huang, S.; Zhao, H.; Zeng, P. Assessing the relationship between leukocyte telomere length and cancer risk/mortality in UK Biobank and TCGA datasets with the genetic risk score and Mendelian randomization approaches. *Front. Genet.* **2020**, *11*, 583106.
10. Cao, X.; Huang, M.; Zhu, M.; Fang, R.; Ma, Z.; Jiang, T.; Dai, J.; Ma, H.; Jin, G.; Shen, H.; et al. Mendelian randomization study of telomere length and lung cancer risk in East Asian population. *Cancer Med.* **2019**, *17*, 7469-7476.
11. Kachuri, L.; Saarela, O.; Bojesen, S.E.; Davey Smith, G.; Liu, G.; Landi, M.T.; Caporaso, N.E.; Christiani, D.C.; Johansson, M.; Panico, S.; et al. Mendelian Randomization and mediation analysis of leukocyte telomere length and risk of lung and head and neck cancers. *Int. J. Epidemiol.* **2019**, *48*, 751-766.
12. Autsavapromporn, N.; Klunklin, P.; Threeratana, C.; Tuntiwechapikul, W.; Hosoda, M.; Tokonami, S. Short telomere length as a biomarker risk of lung cancer development induced by high radon levels: a pilot study. *Int. J. Environ. Res. Public Health* **2018**, *15*, 2152.
13. Doherty, J.A.; Grieshober, L.; Houck, J.R.; Barnett, M.J.; Tapsoba, J.D.; Thornquist, M.; Wang, C.Y.; Goodman, G.E.; Chen, C. Telomere length and lung cancer mortality among heavy smokers. *Cancer Epidemiol. Biomarkers Prev.* **2018**, *27*, 829-837.

14. Haycock, P.C.; Burgess, S.; Nounu, A.; Zheng, J.; Okoli, G.N.; Bowden, J.; Wade, K.H.; Timpson, N.J:; Evans, D.M.; Willeit, P.; et al. Association between telomere length and risk of cancer and non-neoplastic diseases: a Mendelian randomization study. *JAMA Oncol.* **2017**, *3*, 636-651.
15. Rode, L.; Nordestgaard, B.G.; Bojesen, S.E. Long telomeres and cancer risk among 95 568 individuals from the general population. *Int. J. Epidemiol.* **2016**, *45*, 1634-1643.
16. Zhang, C.; Doherty, J.A.; Burgess, S.; Hung, R.J.; Lindström, S.; Kraft, P.; Gong, J.; Amos, C.I.; Sellers, T.A.; Monteiro, A.N.; et al. Genetic determinants of telomere length and risk of common cancers: a Mendelian randomization study. *Hum. Mol. Genet.* **2015**, *24*, 5356-5366.
17. Fernández-Marcelo, T.; Gómez, A.; Pascua, I.; de Juan, C.; Head, J.; Hernando, F.; Jarabo, J.R.; Calatayud, J.; Torres-García, A.J.; Iniesta, P.; Telomere length and telomerase activity in non-small cell lung cancer prognosis: clinical usefulness of a specific telomere status. *J. Exp. Clin. Cancer Res.* **2015**, *34*, 78.
18. Machiela, M.J.; Hsiung, C.A.; Shu, X.O.; Seow, W.J.; Wang, Z.; Matsuo, K.; Hong, Y.C.; Seow, A.; Wu, C.; Hosgood, H.D.; et al. Genetic variants associated with longer telomere length are associated with increased lung cancer risk among never-smoking women in Asia: a report from the female lung cancer consortium in Asia. *Int. J. Cancer* **2015**, *137*, 311-319.
19. Sun, B.; Wang, Y.; Kota, K.; Shi, Y.; Motlak, S.; Makambi, K.; Loffredo, C.A.; Shields, P.G.; Yang, Q.; Harris, C.C.; et al. Telomere length variation: A potential new telomere biomarker for lung cancer risk. *Lung Cancer* **2015**, *88*, 297-303.
20. Sanchez-Espiridion, B.; Chen, M.; Chang, J.Y.; Lu, C.; Chang, D.W.; Roth, J.A.; Wu, X.; Gu, J. Telomere length in peripheral blood leukocytes and lung cancer risk: a large case-control study in Caucasians. *Cancer Res.* **2014**, *74*, 2476-2486.
21. Weischer, M.; Nordestgaard, B.G.; Cawthon, R.M.; Freiberg, J.J.; Tybjærg-Hansen, A.; Bojesen, S.E. Short telomere length, cancer survival, and cancer risk in 47102 individuals. *J. Natl. Cancer Inst.* **2013**, *105*, 459-468.
22. Willeit, P.; Willeit, J.; Mayr, A.; Weger, S.; Oberhollenzer, F.; Brandstätter, A.; Kronenberg, F.; Kiechl, S. Telomere length and risk of incident cancer and cancer mortality. *JAMA* **2010**, *304*, 69-75.
23. Hosgood, H.D.; Cawthon, R.; He, X.; Chanock, S.; Lan, Q. Genetic variation in telomere maintenance genes, telomere length, and lung cancer susceptibility. *Lung Cancer* **2009**, *66*, 157-161.
24. Jang, J.S.; Choi, Y.Y.; Lee, WK.; Choi, J.E.; Cha, S.I.; Kim, Y.J.; Kim, C.H.; Kam, S.; Jung, T.H.; Park, J.Y. Telomere length and the risk of lung cancer. *Cancer Sci.* **2008**, *99*, 1385-1389
25. Wu, X.; Amos, C.I.; Zhu, Y.; Zhao, H.; Grossman, B.H.; Shay, J.W.; Luo, S.; Hong, W.K.; Spitz, M.R. Telomere dysfunction: a potential cancer predisposition factor. *J. Natl. Cancer Inst.* **2003**, *95*, 1211-1218.
